# Impact of aromatase absence on murine intraocular pressure and retinal ganglion cells

**DOI:** 10.1038/s41598-018-21475-x

**Published:** 2018-02-19

**Authors:** Xiaomin Chen, Yang Liu, Yi Zhang, Wendy R. Kam, Louis R. Pasquale, David A. Sullivan

**Affiliations:** 1 Schepens Eye Research Institute, Massachusetts Eye and Ear, and Department of Ophthalmology, Harvard Medical School, Boston, MA USA; 2 Zhongnan Hospital, Wuhan University, Wuhan, China; 3 Affiliated Hospital of Shenzhen University, Shenzhen, China; 4 Channing Division of Network Medicine, Brigham and Women’s Hospital, Harvard Medical School, Boston, MA USA

## Abstract

We hypothesize that aromatase, an enzyme that regulates estrogen production, plays a significant role in the control of intraocular pressure (IOP) and retinal ganglion cells (RGCs). To begin to test our hypothesis, we examined the impact of aromatase absence, which completely eliminates estrogen synthesis, in male and female mice. Studies were performed with adult, age-matched wild type (WT) and aromatase knockout (ArKO) mice. IOP was measured in a masked fashion in both eyes of conscious mice at 12 and 24 weeks of age. Retinas were obtained and processed for RGC counting with a confocal microscope. IOP levels in both 12- and 24-week old female ArKO mice were significantly higher than those of age- and sex-matched WT controls. The mean increase in IOP was 7.9% in the 12-week-, and 19.7% in the 24-week-old mice, respectively. These changes were accompanied by significant 9% and 7% decreases in RGC numbers in the ArKO female mice, relative to controls, at 12- and 24-weeks, respectively. In contrast, aromatase deficiency did not lead to an increased IOP in male mice. There was a significant reduction in RGC counts in the 12-, but not 24-, week-old male ArKO mice, as compared to their age- and sex-matched WT controls. Overall, our findings show that aromatase inhibition in females is associated with elevated IOP and reduced RGC counts.

## Introduction

Several researchers have hypothesized that estrogen deficiency predisposes to optic nerve degeneration^1–3^. This hypothesis is supported by a myriad of observational studies linking estrogen deprivation to an increased risk of open-angle glaucoma^4–7^. Furthermore, variations in several genes involved in estrogen processing are related to open-angle glaucoma^8–10^. Conversely, investigators have also reported that estrogen use may decrease the risk of developing glaucoma^11^, prevent retinal ganglion cell (RGC) death^12,13^, reduce intraocular pressure (IOP^14–17^); preserve visual acuity^12^ and serve as a viable option for treating glaucoma^14,18^.

However, despite these impressive results, there is no global consensus on the role of estrogens in glaucoma. Indeed, there is controversy. There is no robust evidence indicating an overall sexual predilection for OAG^18,19^. Tamoxifen, an estrogen receptor blocker that is widely used to treat breast cancer, does not appear to be associated with increased risk of glaucoma^20^. Abramov and colleagues found no difference in IOP among postmenopausal women with a history of hormone replacement therapy versus women who did not use postmenopausal hormones^21^. No significant correlation between IOP level and serum estradiol was found among 62 postmenopausal women, 30 of which were using hormone replacement therapy^22^. Furthermore another study from India did not find relations between female reproductive factors and open-angle glaucoma^23^.

These discrepant findings may be attributed, at least in part, to variations in experimental design, including differences in the age, sex, and endocrine status of subjects, as well as in the dosage and time course of estrogen therapy, and even in the methods of analysis. Nevertheless, it is extremely important to determine whether estrogen deficiency promotes, and estrogen treatment suppresses, IOP elevation, RGC death and optic neuropathy. The reason is that estrogen dynamics may contribute significantly to the development of primary open-angle glaucoma in women^2^ and possibly men^9^. Furthermore, there is an ever-increasing use of aromatase inhibitors for the treatment of breast and ovarian cancer in postmenopausal women. These inhibitors prevent the biosynthesis of estrogens and could possibly enhance the risk and/or severity of glaucoma^24^.

**Figure 1:**
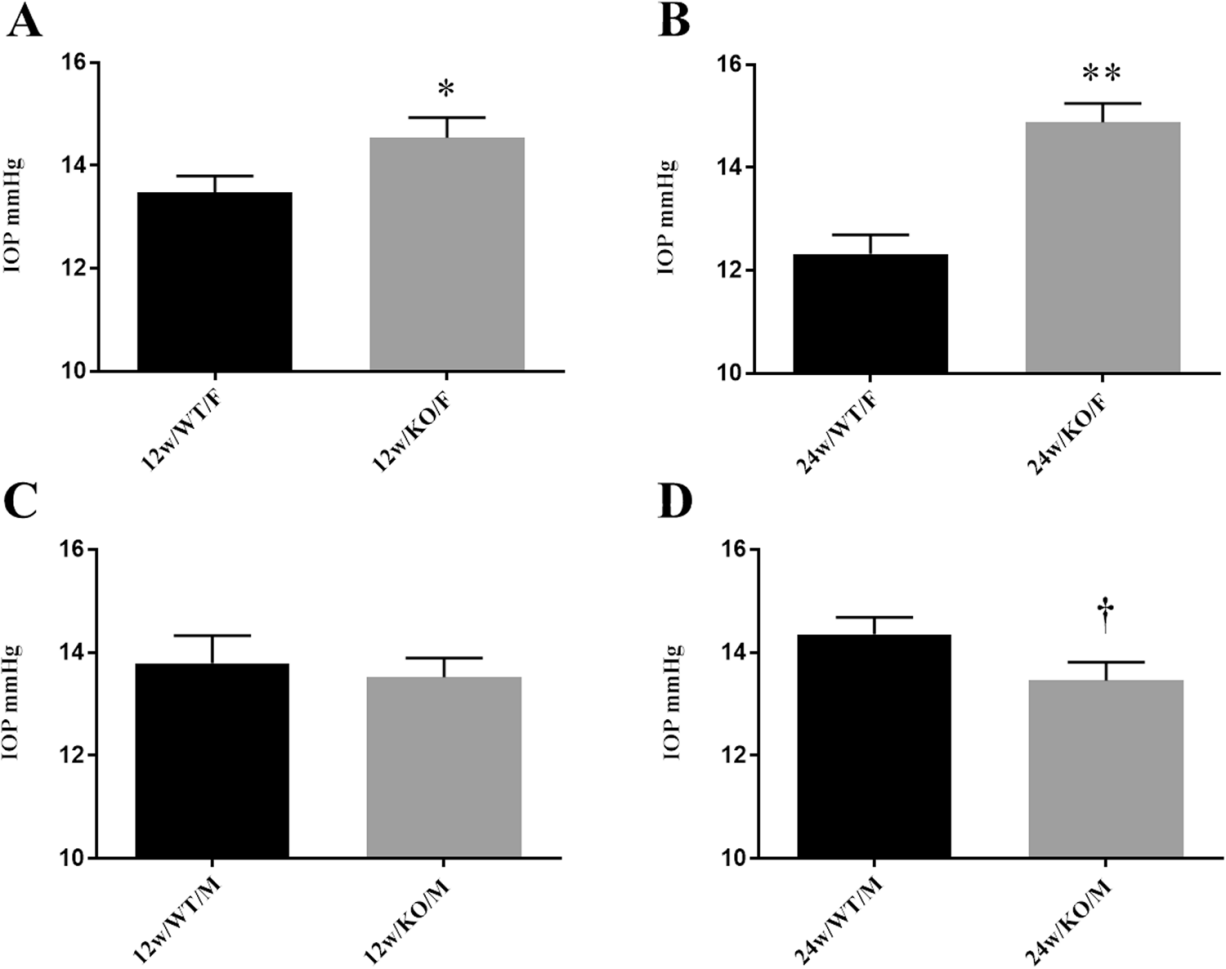
Influence of complete aromatase absence on the IOP in 12- and 24-week old mice. Columns represent the mean ± SE. Abbreviations: 12w-12 week; 24w-24 week; WT-wild type; KO-knockout; F-female; M-male. Significantly less (p < 0.05; *p < 0.001**) or greater (<0.05)^†^ than WT control.

We used C57BL/6 J - aromatase knockout (ArKO) mice to begin to clarify whether estrogen deprivation is associated with glaucomatous features. The ArKO mice were generated by the targeted disruption of exon IX in the *cyp19* gene and lack aromatase activity^25^. Aromatase is a cytochrome P450 enzyme that catalyzes the formation of estrogens from androgens^26–28^, and contributes to a number of sex-specific differences throughout the body^29–33^. In the absence of aromatase, the synthesis of estrogens is completely eliminated^34–36^. Accordingly, we examined whether female ArKO mice exhibit heightened IOP and RGC loss, as compared to their wildtype (WT) controls. For comparison, we also evaluated male ArKO mice. (Figure 1).

## Results

### IOP levels in female and male ArKO mice

To determine whether aromatase absence influences IOP, we measured IOP levels in both eyes of 12- and 24-week old female and male WT and ArKO mice (n = 8/group). Our results demonstrate that IOP levels in both 12- (WT = 13.5 ± 0.3; ArKO = 14.5 ± 0.4, p < 0.05) and 24- (WT = 12.3 ± 0.4; ArKO = 14.9 ± 0.4, p < 0.001) week old female ArKO mice were significantly higher than those of age- and sex-matched WT controls (Fig. 1). The mean increases in IOP ranged from 7.9% (1.1 mmHg) in the 12-week-, to 19.7% (2.6 mmHg) in the 24-week-old mice, respectively.

**Figure 2:**
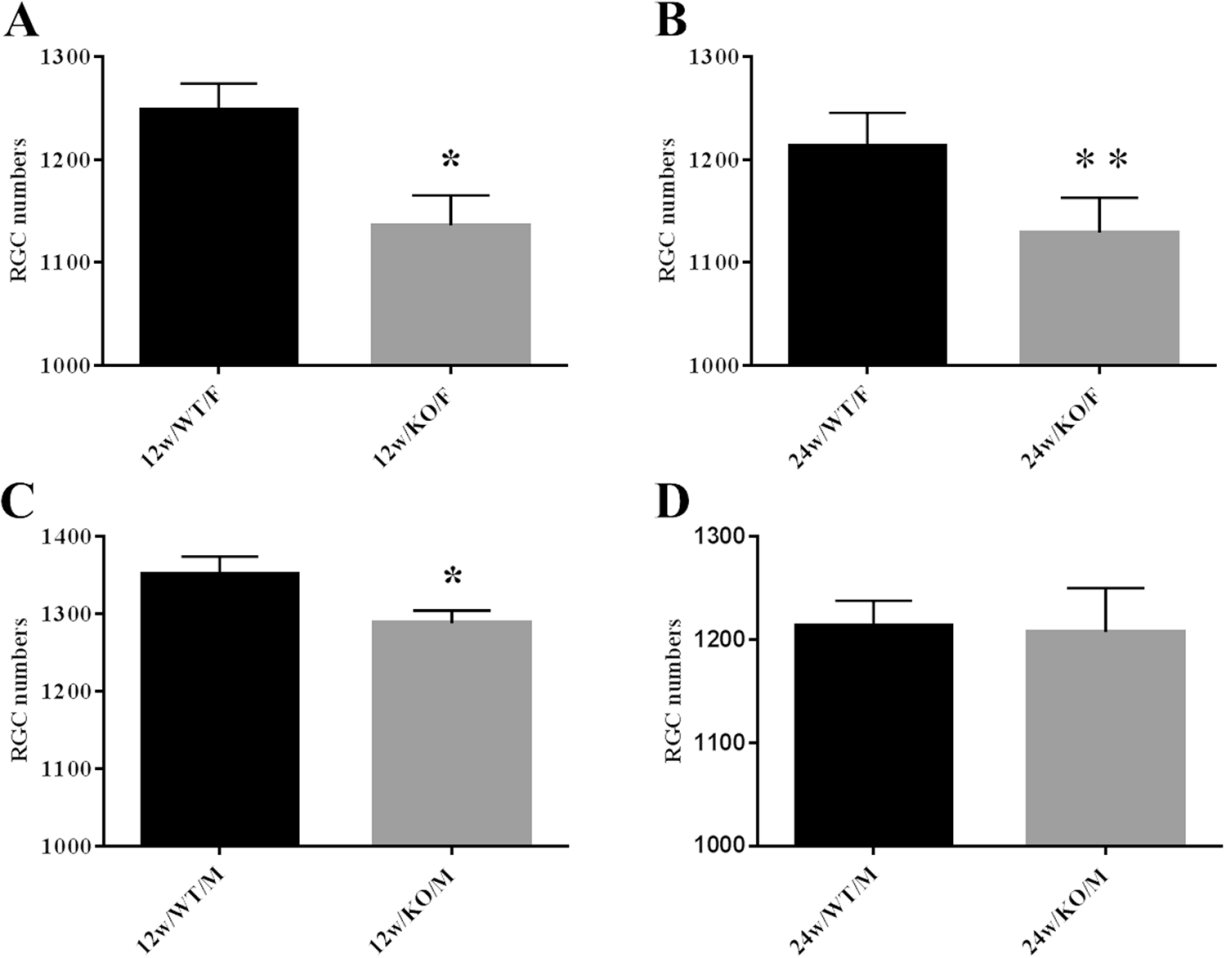
Impact of complete aromatase absence on the RGC count in 12- and 24-week old mice. Columns represent the mean ± SE. Abbreviations: 12w-12 week; 24w-24 week; WT-wild type; KO-knockout; F-female; M-male. Significantly less (p < 0.05)* or (p < 0.05, one-tail)** than WT control.

In contrast, aromatase deficiency did not lead to increases in the IOP of 12- (WT = 13.8 ± 0.5; ArKO = 13.5 ± 0.4) or 24- (WT = 14.4 ± 0.3; ArKO = 13.5 ± 0.4) week old male ArKO mice, as compared to WT controls. Rather, the lack of aromatase was associated with a significant decrease in the IOP of 24-week old male mice (p < 0.05; Fig. 1).

Our findings related to the influence of aromatase were the same if we limited IOP comparisons to the same (e.g. right vs. right) or to the opposite (e.g. right vs. left) eyes of sex- and age-matched WT vs ArKO mice (data not shown). There were no significant differences in IOP levels between the left and right eyes of WT mice, or between left and right eyes of ArKO mice. In addition, there was no significant difference between the IOP of 12- vs. 24-week old female or male ArKO mice.

Our IOP data are based upon the analyses of one mean IOP per mouse (i.e. average of 12 IOP values/mouse, originating from 3 values/eye/day, two eyes/mouse, on 2 consecutive days).

#### RGC counts in female and male ArKO mice

To examine the effect of complete aromatase absence on RGC counts, we analyzed RGC numbers in retinas of 12- and 24-week old female and male WT and ArKO mice (n = 8/group). As shown in Fig. 2, RGC numbers in the ArKO female mice were significantly reduced, as compared to age- and sex-matched WT controls (12 week old, WT = 1,249 ± 25; ArKO = 1,136 ± 29, p = 0.012; 24 week old, WT = 1,213 ± 33; ArKO = 1,129 ± 34, p = 0.049, one tail). Similarly, there was a significant decrease in RGC counts in the 12-week old male ArKO mice, relative to their age- and sex-matched WT controls (WT = 1,351 ± 24; ArKO = 1,288 ± 16, p = 0.047). No significant difference existed between the RGC numbers in 24-week-old male WT and male ArKO mice (WT = 1,214 ± 24; ArKO = 1,208 ± 43). Furthermore, there was no significant difference between the RGC counts of 12- vs. 24-week old female ArKO mice or 12- vs. 24-week old male ArKO mice (Fig. 2).

**Figure 3:**
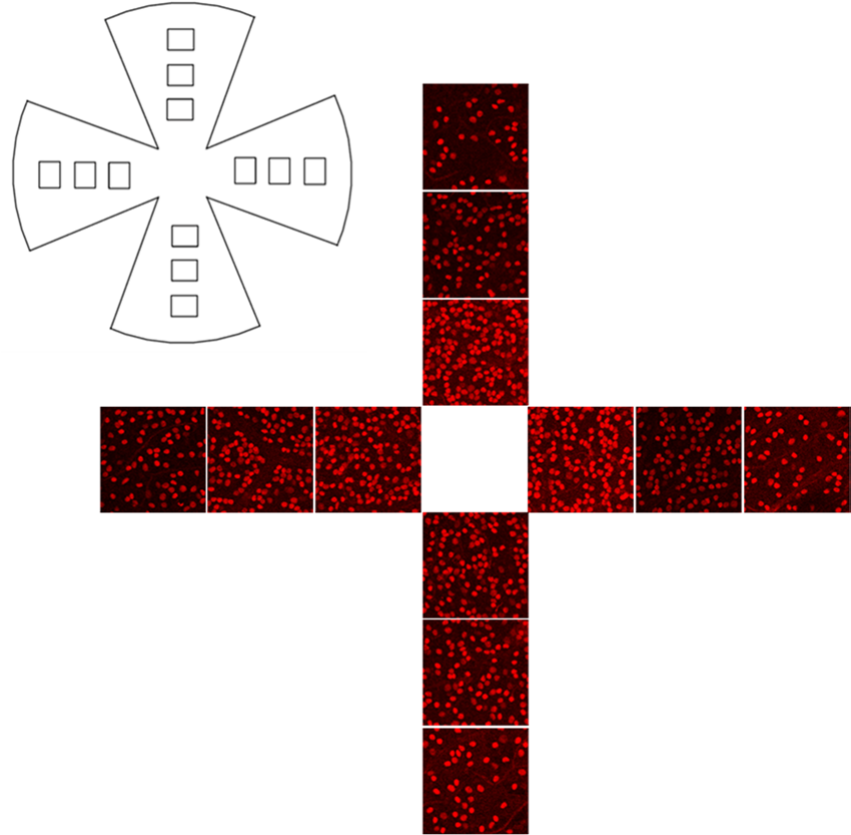
Immunohistochemical identification of mouse RGCs. Retinal samples were mounted and RGCs imaged with a confocal microscope. Cells were counted manually in 3 random areas along the centerline of each quadrant (total = 3 counts/quadrant, 12 counts/retina). RGCs were clearly visible by using a primary anti-Brn3a antibody and a secondary anti-IgG antibody.

## Discussion

Glaucoma is the second leading cause of blindness in the world and is a slowly progressing neurodegenerative disease^37,38^. It is characterized by a gradual loss of RGCs, which leads to a loss of vision^37,39,40^. The most common form of glaucoma, occurring in 70 to 90% of patients, is primary open angle glaucoma (POAG)^41–43^.

In one epidemiological study, women had a significantly lower incidence of POAG, as compared to men, until the age of 80 years^44^. This sex-related difference has been linked to the extent of lifetime estrogen exposure. Indeed, there is an association between increased estrogen exposure and a reduced POAG risk^37^. Conversely, studies have shown that a decreased exposure (i.e. early loss of estrogens), due to late menarche, oral contraceptive use, early menopause, and a shorter duration between menarche to menopause, is associated with an increased risk of POAG^5–7,37,45^. Given this background, we assessed glaucoma features in female ArKO mice.

Our present study demonstrates that aromatase absence is associated with a significant increase in the IOP levels of 12- and 24-week old female ArKO mice. The extent of this IOP difference (i.e. 1.1 to 2.6 mmHg) is comparable to that (i.e. 0.5 to 0.6 mmHg) found between postmenopausal women taking, versus not taking, estrogen replacement therapy as part of a post-hoc analysis of a randomized clinical trial^17^. In addition, the aromatase deficiency in ArKO mice is associated with a significant decrease in RGC counts. Our findings support the myriad of human data linking estrogen deprivation to the development of glaucoma among women.

In contrast to the responses of ArKO females, aromatase deficiency did not lead to an increased IOP in male mice. There was, though, a significant decrease in RGC counts in the 12-, but not 24-, week-old male ArKO mice, as compared to their age- and sex-matched WT controls. This sexually dimorphic response to complete aromatase absence is not surprising. We have previously found that almost all genes regulated by aromatase in non-retinal ocular tissues (i.e. meibomian and lacrimal glands) are sex-specific, and thus different, in male and female mice^46,47^. Similar sex-related differences have also been found in the liver, bone, hematopoietic microenvironment, adipose tissue and brain^30,48–55^. These variations between male and female responses may reflect the loss of estrogens, reduced serum adiponectin^56^, alterations in neural activity^57,58^, as well as the heightened serum levels of testosterone, androstenedione, prolactin, follicle-stimulating hormone, luteinizing hormone (LH), insulin-like growth factor 1 (IGF-1) and leptin in ArKO mice^25,50,55,56,59–61^, some hormone-independent processes^62,63^, and the influence of Y-linked genes or X inactivation in the ArKO strain^64–67^. The fact that aromatase deficiency does impact the male eye is consistent with studies demonstrating that aromatase and estrogen actions are very important in other male tissues^52–55,68–70^.

There are several specific ways in which estrogen may directly affect vulnerability to glaucoma. One mechanism may involve estrogen binding to saturable, high affinity, and steroid-specific estrogen receptors (ERs) in retinal tissues, especially in RGCs. ERs, which are members of the nuclear receptor superfamily of ligand-inducible transcription factors, mediate most of the actions of estrogens throughout the body^71^. Estrogens have two nuclear ERs, termed α and β, which are highly homologous in their DNA and ligand binding domains, are often differentially distributed, and which direct different, and sometimes opposite, functions of estrogens^71–80^. After estrogen binds to the ER, the activated hormone-receptor complex typically associates with estrogen responsive elements in the regulatory region of specific target genes and, in combination with appropriate co-activators and enhancers, modulates gene transcription, protein synthesis and tissue function^73^. Consistent with this mechanism, we and others have identified ER mRNA and protein in the retina, as well as ERα and ERβ in RGCs^81–83^. By acting through these nuclear receptors, estrogens might then influence, for example, vascular resistance and the perfusion of the optic nerve, RGCs, and their supporting structures^1,84,85^.

A second mechanism to explain estrogen effects on the eye may also involve plasma membrane ERs. These pathways, which typically signal within seconds to minutes, involve estrogen interaction with stereospecific membrane ERs and lead to rapid changes in membrane fluidity, the activity of neurotransmitter receptors and/or the control of transcription factors^86–92^. We have identified membrane ERs in other ocular tissues^93^. It is also possible that estrogen influence could be modified by the activity of retinal steroidogenic enzymes.

A third mechanism by which estrogens impact glaucoma may be operative in humans, but not mice, and involve the intracrine synthesis of estrogens. Unlike lower mammals (e.g., mice), in which the ovaries and testes are the primary origin of active sex steroids^94,95^, most biologically active sex steroids in women do not originate from the ovary and do not circulate in the blood. The vast majority of estrogens (i.e. 75% before, and 100% after, menopause), and almost all androgens, are synthesized in peripheral tissues from dehydroepiandrosterone (DHEA)^3,94–99^. Indeed, humans and primates are unique in possessing adrenal glands that secrete large amounts of DHEA, which is then converted into androgens and estrogens by steroidogenic enzymes in peripheral sites. This hormonal process, termed intracrinology, allows target tissues to adjust the formation and metabolism of sex steroids to local requirements^95,97^. After intracrine synthesis and local action, sex steroids are metabolized and released into the circulation. The critical steroidogenic enzymes involved in estrogen synthesis and metabolism all exist and are active in the retina^3,100–102^.

A number of other processes may also be involved in altering the IOP and RGC counts in female ArKO mice. First, the increased serum leptin concentrations in ArKO mice^50^ may counteract the glaucomatous effect of estrogen deficiency, given that leptin is thought to be a neuroprotective agent^103^. Such leptin action might contribute to the lack of progressive IOP and RGC changes in female ArKO mice from 12 to 24 weeks of age. Second, changes in adiponectin^56^, IGF-1^55^ and LH^61^ levels may affect glaucoma progress^104^, RGC survival^105^ and retinal dynamics^106^, respectively. The obesity^107,50^ and glucose intolerance^108^ in these ArKO mice may lead to the development of diabetes^109^, increased IOP^110,111^ and glaucoma^112^. Third, the decreased blood pressure in female ArKO mice^113^ may promote glaucomatous changes^114^.

There are limitations in our study. We did not assess the retinal functional consequences in the ArKO mice. We did not extend phenotype observations beyond 24 weeks. Furthermore, we did not explore the mechanism for IOP increase or RGC dropout in female mice, nor the compensatory mechanisms that might have produced minimal or no glaucomatous features in male ArKO mice.

Overall, our results demonstrate that aromatase absence produces a glaucoma phenotype that is more significant in female mice. Our study is a reverse engineering approach to model one aspect of POAG and to support the epidemiological literature suggesting that relative estrogen deficiency increases the risk of POAG, which is a multifactorial disease. Therefore, it is not surprising that the IOP increases and RGC losses were modest and relatively sex-specific. We are not modeling nonspecific end organ optic nerve damage, but rather a complex disease (POAG) by precisely altering one of the many exposures involved in disease pathogenesis. In this regard our findings are very important, because the modest effects on IOP and the RGCs are consistent with epidemiological data showing modest reductions in IOP and in glaucoma risk related to estrogen exposure^1,72^.

## Methods

### Mice

We obtained breeding pairs of C57BL/6 J - aromatase knockout (ArKO) heterozygous mice from Dr. Nabil J. Alkayed (Knight Cardiovascular Institute, Oregon Health & Science University, Portland, OR), who, in turn, had procured them from Dr. Orhan Oz (University of Texas Southwestern Medical Center, Dallas, TX). Animals were shipped to Charles River Breeding Laboratories (Wilmington, MA) for initial quarantine, health monitoring and serology, and then forwarded to the Animal Facilities of the Schepens Eye Research Institute (SERI). Mice were housed and bred in constant temperature rooms with fixed light/dark intervals of 12 hours duration. All mice were fed the PicoLab Rodent Diet 20 (#5053; LabDiet, St Louis, MO).

We generated hundreds of WT, ArKO and heterozygous mice for these studies. Twenty one-day-old offspring were genotyped according to modifications of a published protocol^25^. In brief, genomic DNA was isolated from ear punches by using a GenEluteTM Mammalian Genomic DNA Miniprep Kit (Sigma-Aldrich). The DNA was amplified by PCR with a Hybaid OMN-E thermocycler (Thermo Electron Corp) by using exon 9 gene primers (forward: GTGACAGAGACATAAAGATCG; reverse: GTAAATTCATTGGGCTTAGGG) and *neo* gene primers (forward: ATCAGGATGATCTGGACGAAGA; reverse: CCACAGTCGATGAATCCAGAA). The PCR conditions were 1 cycle (3 minutes at 94 °C), 31 cycles (40 second at 94 °C, 30 second at 55 °C, 45 second at 72 °C) and 1 cycle (5 minutes at 72 °C) and amplicons were evaluated on 2.5% agarose gels. Band sizes were 220 bp from WT mice, 170 bp from ArKO mice, and both fragments from heterozygotes. After mice were sacrificed, we repeated the genotyping to confirm the genetic background.

All experiments with these mice were approved by the SERI Institutional Animal Care and Use Committee (IACUC) and adhere to the Association for Research in Vision and Ophthalmology Statement for the Use of Animals in Ophthalmic and Vision Research.

### IOP measurements

Measurements of IOP were performed on conscious mice. We developed procedures to measure IOP (n = 6 consecutive measurements per IOP value, 3 values/eye/day, 2 consecutive days) at the central cornea of awake mice with a TonoLab tonometer (Icare, Tampere, Finland). We secured mice in DecapiCone bags, which calms the animals and also prevents head movement. Measurements of the IOP were performed in a masked fashion between 10 and 11 AM. We did not use anesthesia, because such exposure is known to significantly alter IOP^115^.

We tested our procedures on 5 mice for three consecutive days to determine whether adaptation to the apparatus and examination process was required. Our results showed that an adaptation period is not necessary. IOP measurements in the right and left eyes remained consistent in a given mouse, and typically varied little from day to day. The mean IOP variation in right and left eyes of the WT and ArKO mice from the first to second days of IOP measurements is shown in Table 1.

**Table 1:** Variation in mean IOP levels in right and left eyes of mice from days 1 to 2 of IOP measurements.

Age (weeks)	Genotype	Sex	Right eye (mmHg)	Left eye (mmHg)
12	WT	F	−0.1	−0.3
12	KO	F	−0.3	−0.7
12	WT	M	+0.8	+0.6
12	KO	M	−0.8	−1.5
24	WT	F	−0.7	+0.3
24	KO	F	+1.0	+0.5
24	WT	M	−1.3	−1.7
24	KO	M	+0.6	+1.1

IOP levels (n = 6 consecutive measurements per IOP value, 3 values/eye/day, 2 consecutive days) were recorded at the central cornea of awake mice. The difference between the mean IOP values on Days 1 and 2 in the right and left eyes of female and male WT and ArKO mice (n = 8/mice group) at 12 and 24 weeks of age are shown. The “−” symbol stands for “negative” (i.e. the group mean IOP value decreased from Day 1 to 2 by that amount). The “+” symbol stands for “positive” (i.e. the group mean IOP value increased from Day 1 to 2 by that amount).

### RGC counting

At the termination of experiments, mice were sacrificed by CO_2_ inhalation, tissue was obtained to confirm genotype, and the vasculature was irrigated with phosphate-buffered saline (PBS). Eyes were removed, fixed in 4% paraformaldehyde for 2 hours, and then processed for the preparation of retinal flat mounts (cut into 4 quadrants^116,117^). Retinal samples were permeated with 0.5% Triton X-100 for 15 minutes at −80 °C, exposed to mouse anti-mouse Brn3a antibody (Millipore; diluted 1:200 in blocking buffer [2% bovine serum albumin and 2% Triton]) overnight at 4 °C, then incubated with donkey anti-mouse IgG antibody (Millipore; diluted 1:200 in blocking buffer) for 2 hours at room temperature. The use of the mouse anti-mouse Brn3a antibody for the identification of RGCs in mouse retinal sections has previously been described^118,119^. Samples were then mounted and RGCs were imaged with a confocal microscope (Leica TCS SP5 CLSM, zoom = 800 fold). RGCs were counted manually in 3 random areas along the centerline of each quadrant (total = 3 counts/quadrant, 12 counts/retina). More specifically, we used the optic nerve head as the origin with 3 standard regions distributed at 1-mm intervals along the central line of each quadrant. The area of each region at 800x magnification equaled 0.037 mm^2^. This analytical approach permitted us to account for retinal eccentricity. Overall, we optimized the techniques for the identification of mouse RGCs (Fig. 3).

### Statistical analyses

Normality of the data was confirmed by using the Shapiro-Wilk and Kolmogorov-Smirnov tests. Unless otherwise noted, IOP and RGC comparisons were performed with two-tailed Student’s t-tests. A P < 0.05 was considered significant. Data are provided as the mean and standard error. All statistical analyses were performed with SPSS (IBM, Armonk, New York) and/or Prism 7 (GraphPad Software, Inc., La Jolla, CA).

### Data availability statement

All data generated or analyzed during this study are included in this published article.
